# Influence of Post-Treatment Operations on Structural Properties and Photocatalytic Activity of Octahedral Anatase Titania Particles Prepared by an Ultrasonication-Hydrothermal Reaction

**DOI:** 10.3390/molecules191219573

**Published:** 2014-11-26

**Authors:** Zhishun Wei, Ewa Kowalska, Bunsho Ohtani

**Affiliations:** Catalysis Research Center, Hokkaido University, Sapporo 001-0021, Japan; E-Mails: wei@cat.hokudai.ac.jp (Z.W.); ohtani@cat.hokudai.ac.jp (B.O.)

**Keywords:** titania, photocatalytic activity, octahedral anatase particles, post-treatments, time-resolved microwave conductivity

## Abstract

The influence of changes in structural and physical properties on the photocatalytic activity of octahedral anatase particles (OAPs), exposing eight equivalent {101} facets, caused by calcination (2 h) in air or grinding (1 h) in an agate mortar was studied with samples prepared by ultrasonication (US; 1 h)–hydrothermal reaction (HT; 24 h, 433 K). Calcination in air at temperatures up to 1173 K induced particle shape changes, evaluated by aspect ratio (AR; *d*_001_/*d*_101_ = depth vertical to anatase {001} and {101} facets estimated by the Scherrer equation with data obtained from X-ray diffraction (XRD) patterns) and content of OAP and semi-OAP particles, without transformation into rutile. AR and OAP content, as well as specific surface area (SSA), were almost unchanged by calcination at temperatures up to 673 K and were then decreased by elevating the calcination temperature, suggesting that calcination at a higher temperature caused dull-edging and particle sintering, the latter also being supported by the analysis of particle size using XRD patterns and scanning electron microscopic (SEM) images. Time-resolved microwave conductivity (TRMC) showed that the maximum signal intensity (*I*_max_), corresponding to a product of charge-carrier density and mobility, and signal-decay rate, presumably corresponding to reactivity of charge carriers, were increased with increase in AR, suggesting higher photocatalytic activity of OAPs than that of dull-edged particles. Grinding also decreased the AR, indicating the formation of dull-edged particles. The original non-treated samples showed activities in the oxidative decomposition of acetic acid (CO_2_ system) and dehydrogenation of methanol (H_2_ system) comparable to and lower than those of a commercial anatase titania (Showa Denko Ceramics FP-6), respectively. The activities of calcined and ground samples for the CO_2_ system and H_2_ system showed almost linear relations with AR and *I*_max_, respectively, suggesting that those activities may depend on different properties.

## 1. Introduction

Titanium(IV) oxide (titania) has been the most frequently used photocatalyst in various areas [1,2]. In almost all practical applications of photocatalysis, titania has been used as a photocatalyst because of its many advantages including low price, high photostability, nontoxicity and superior redox ability [3]. Although studies on titania photocatalysis [3] have suggested the desired structural and/or physical properties for high-level photocatalytic activity, e.g., anatase crystallites rather than rutile ones and smaller particles, *i.e.*, higher specific surface area, intrinsic interpretation for possible structure-activity correlations has not yet been obtained, at least to the authors’ knowledge [4,5,6].

Particle morphology has recently attracted much attention from scientists as a possible key parameter for controlling the activity of photocatalyst particles [7,8,9,10,11,12,13,14]. Various methodologies have been developed to control particle morphology by using diverse treatments such as ultrasonication, grinding, washing, microwave irradiation, and thermal and pressure treatments [15,16,17], and by selective preparation of single-crystalline facetted photocatalyst particles [18,19,20]. Along this line, octahedral anatase particles (OAPs), exposing eight equivalent {101} facets, with sizes of several tens of nanometers, have been prepared by ultrasonication (US) of partially proton-exchanged potassium titanate nanowires followed by hydrothermal reaction (HT) [21,22]. As is suggested by the fact that natural anatase titania minerals have been found in an octahedral shape, the {101} facets are thermodynamically most stable and tend to be exposed when titania is prepared under equilibrium conditions such as HT. In the previous study of our group, it was shown that OAPs prepared by HT after US showed relatively high activity for oxidative decomposition of acetic acid under an aerobic atmosphere (CO_2_ system) but low activity for methanol dehydrogenation under an argon atmosphere (H_2_ system) [21,22,23].

As has been often and commonly observed for particulate photocatalysts, photocatalytic activities of OAP-containing samples prepared under different US and HT conditions have different physical/structural properties, morphologies and photocatalytic activities. However, it was accidentally observed that particles with almost the same structural activities, such as specific surface area, crystalline composition and particle size, except only for OAP content, could be prepared by changing only US time, and the photocatalytic activity, especially for a CO_2_ system, was almost proportional to the OAP content; *i.e.*, the morphology of particles directly determines the photocatalytic activity [23].

In the present study, an OAP-containing sample was calcined or ground in an agate mortar in air to change mainly the particle morphology, and the influence of change in morphology on photocatalytic activity was examined in order to clarify the intrinsic reason for the morphology-dependent photocatalytic activity of anatase titania photocatalyst particles.

## 2. Results and Discussion

### 2.1. Preparation of Original OAP-Containing Sample

**Figure 1:**
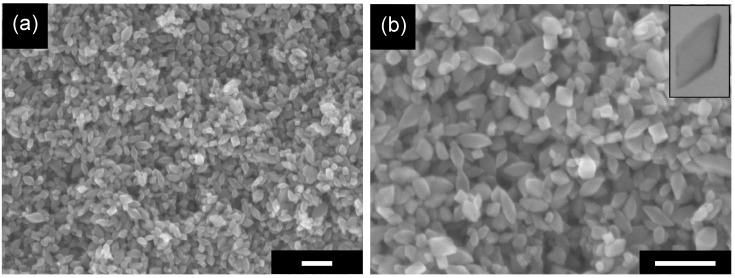
SEM images with (**a**) low magnification and (**b**) high magnification of the original OAP-containing sample. Scale bar: 100 nm.

**Figure 2:**
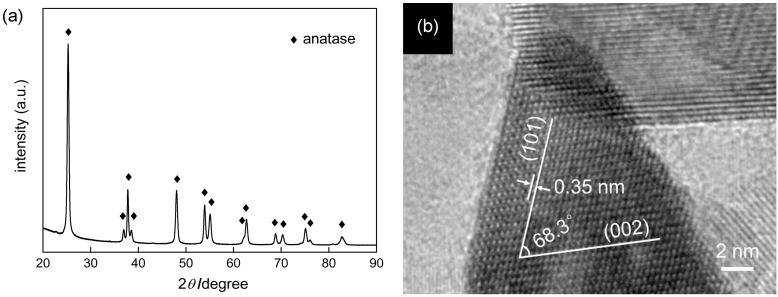
(**a**) XRD pattern and (**b**) HRTEM image of the original OAP-containing sample.

The previously reported US-HT process [21] led to conversion of partially protonated potassium titanate nanowires (TNWs) into anatase titania particles of predominantly octahedral shape. SEM images of the original sample (without post-treatment) are shown in Figure 1. The obtained particles were smaller than 50 nm and exhibited various morphologies, *i.e.*, OAP, semi-OAP and others (see the Experimental Section). In the XRD pattern, only peaks assigned to anatase crystallites appeared (Figure 2a). Images of high-resolution transmission electron microscopy (HRTEM) supported the presence of single crystals of anatase as shown in Figure 2b; *i.e.*, lattice fringes with a spacing of 0.35 nm and an angle between two kinds of fringes of 68.3° agreed well with the previously reported (101) lattice spacing and angle between (101) and (001) [21].

### 2.2. Influence of Calcination on the Structure of Particles

**Figure 3:**
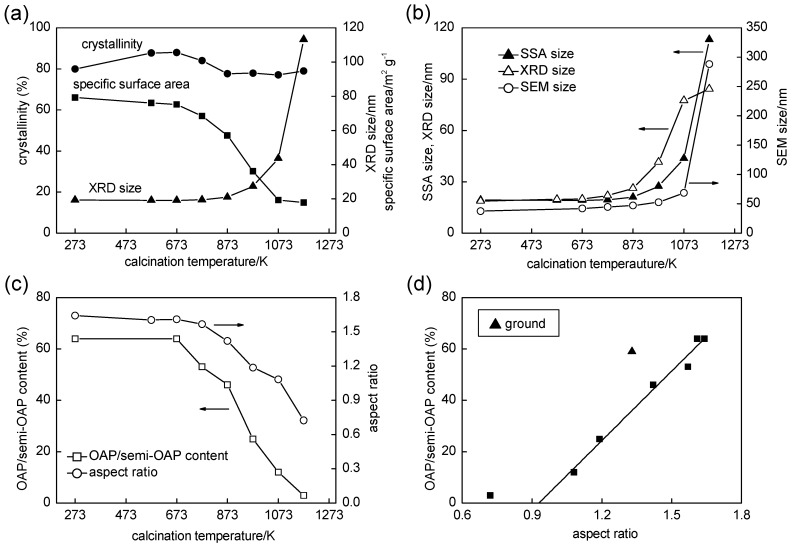
(**a**) Correlations between crystalline size (XRD size), crystallinity, specific surface area and calcination temperature; (**b**) Comparison of XRD size, SEM size and SSA size of samples as functions of calcination temperature; (**c**) Changes in OAP/semi-OAP content and aspect ratio (AR) as functions of calcination temperature; (**d**) OAP/semi-OAP content as a function of AR.

The influence of calcination on the structural properties of samples was studied by changing calcination temperatures (573–1173 K; 2 h) and keeping the other process conditions, *i.e.*, HT time (24 h), HT temperature (433 K), US time (1 h), TNW amount of titanate nanowires (267 mg) and Milli-Q water volume (80 mL) the same. Figure 3a shows the influence of calcination temperature on crystallinity (anatase; content of crystalline phases) and specific surface area (SSA). It should be noted that no peaks assigned to rutile crystalline were observed even with calcination at 1173 K. This feature will be discussed later. Figure 3b shows changes in crystallite size estimated from a 101 XRD peak (“XRD size”; same as in Figure 3a), particle size estimated by SEM observation (“SEM size”), and particle size expected from SSA (“SSA size”) calculated with the assumption of spherical uniform-sized anatase particles [4]. It is expected that SEM size, the longest part of each particle, may be larger than XRD size, depth of the particle measured in the direction vertical to the {101} lattice plane. Furthermore, SEM size may be the size of aggregated particles if they are seen as a single crystal, while XRD size corresponds to the average size of a single crystalline part in aggregated particles. Considering those problems, use of XRD size seems better to discuss the change in particle size. As seen in Figure 3a, with elevation of calcination temperature, XRD size slightly increased at ≤973 K but was much larger in the temperature range higher than 973 K, while SSA decreased even at a lower temperature, *i.e.*, 773 K. Therefore, at a temperature higher than 773 K, a difference between XRD size and SSA size can be seen in Figure 3b, indicating partial sintering, with lattice mismatch, *i.e.*, formation of grain boundaries, of anatase crystallites by calcination. The decrease in crystallinity in the temperature range of 673–873 K is attributable to the formation of grain boundaries. (The increase in crystallinity at the lower temperature might be due to dehydration and/or crystallization of amorphous phase.) Thus, calcination at temperatures of 773–1073 K in air induced sintering of some of the particles without lattice matching as the difference between XRD size and SSA size shows; XRD size became comparable to or larger than that of SSA size by calcination at 1173 K, suggesting fusion of crystallites to a larger single crystal.

These findings indicate that the original OAP-containing particles (content of OAP and semi-OAP: 62%) are stable toward heat-induced crystal transformation at the temperature at lowest below 1073 K, presumably due to their exposure of ordered {101} facets with less defects, which may trigger crystal transformation into larger single crystals (fusion) or rutile. As was stated above, all of the samples, even those heated at 1173 K, reported here included no rutile phase, though it has been reported that conversion of anatase to rutile usually occurs at much lower temperatures, e.g., 823–973 K [24,25,26,27]. The high level of heat tolerance of the present OAP-containing particles is attributable to the above-mentioned particle morphology exposing ordered facets. It has been reported that rice-like titania nanorods were only stable enough to convert 2% of anatase into rutile at 1173 K [28]. Another possible reason for the heat tolerance is the presence of stabilizers, *i.e.*, potassium cations possibly contaminated from TNWs. It was reported that the presence of impurities enhanced the heat tolerance of anatase; e.g., lanthanum(III) oxide-modified titania underwent phase transformation by calcination at >1073 K [29]. Study on this heat tolerance is now under way and the details will be published elsewhere [30].

**Figure 4:**
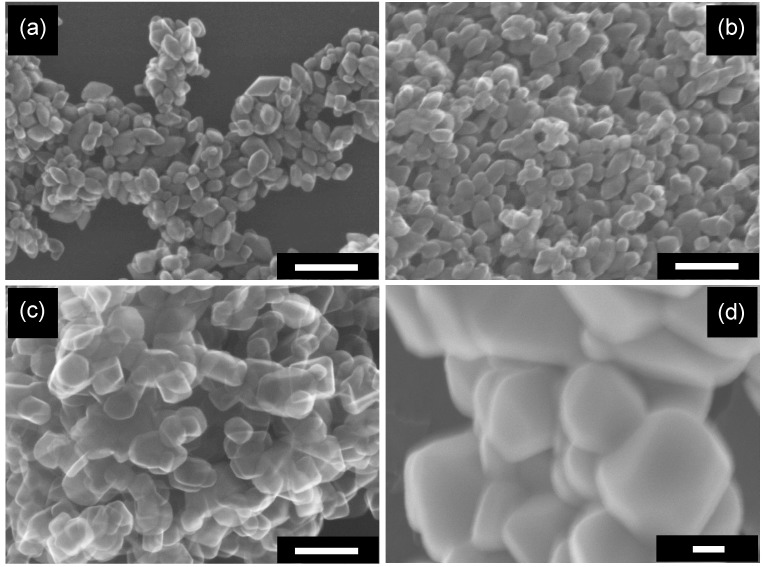
SEM images of OAPs with calcination temperatures of (**a**) 673 K; (**b**) 873 K; (**c**) 1073 K and (**d**) 1173 K. Scale bar: 100 nm.

Calcination also changed the morphology of samples. As Figure 4 shows, sharply angulate particles became round-shaped particles by calcination accompanied by the above-mentioned sintering, and the higher the calcination temperature was, the higher was the extent of round edging. These observations were quantified using OAP (and semi-OAP) content and aspect ratio (AR) as shown in Figure 3c. The former was evaluated by counting numbers of OAPs, semi-OAPs and others in SEM images, and the latter was evaluated as the ratio of crystallite sizes calculated from 004 and 101 XRD-peak widths and might be closely related to the OAP content. With calcination at < 675 K, there was little change in either OAP content or AR, *i.e.*, morphology was not changed in this low temperature region. This is consistent with results of the above-mentioned particle-size analysis. In a higher temperature region, ≥773 K, both OAP content and AR were decreased to 3% and 0.72 at 1173 K, respectively, suggesting that sintering (or fusion) of crystallites induced a change in morphology to a round-edged shape. As Figure 3d shows, OAP and semi-OAP contents decreased linearly with decrease in AR > 1, indicating that both are measures of particle shape; the content of semi-OAPs, which include particles with round-edged summits, increased with decrease in AR. The x-intercept of the extrapolated linear part of plots was *ca*. 0.9. Based on the assumption that an octahedral particle is changed to a decahedral particle that exposes eight {101} and two {001} facets, the AR value giving the lowest surface area/volume (weight) ratio is estimated to be ca. 0.95 [31], similar to the observed intercept. It seems that the calcination-induced AR decrease is accounted for by modification of particle shape to give smallest surface area/volume ratio. Considering the arbitrary property of OAP content, AR will be used when discussing shape dependence in the following sections.

### 2.3. Influence of Calcination on Photocatalytic Activities

**Figure 5:**
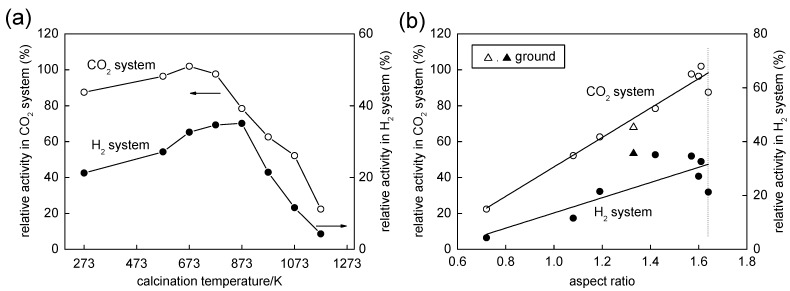
(**a**) Correlation between photocatalytic activity and calcination temperature (data of the original uncalcined sample shown at “273 K”); (**b**) Photocatalytic activities as functions of aspect ratio (AR). Plots for the original uncalcined sample are on a dotted line. Open (CO_2_) and closed (H_2_) triangles are of the ground sample.

Photocatalytic activity for two reaction systems, *i.e.*, decomposition of acetic acid (CO_2_ system) and methanol dehydrogenation (H_2_ system), was examined. The dependence of photocatalytic activities on calcination temperature is shown in Figure 5a. The highest photocatalytic activity in the CO_2_ system was obtained for the sample calcined at 673 K. The influence of calcination was less evident in the H_2_ system than in the CO_2_ system, and the sample calcined at 873 K showed 1.7 times higher photocatalytic activity than that of the original uncalcined sample. Although this activity trend can be explained, in a conventional manner, by a good balance of higher specific surface area and higher crystallinity [32], such an explanation does not give any intrinsic insights into the correlation between the physical/structural property and photocatalytic activity. In a previous study on photocatalytic activity of OAP-containing particles, it was found that photocatalytic activities for CO_2_ and H_2_ systems are governed only by the OAP content of samples; the activities were linearly increased with increase in OAP content for samples with almost the same other structural properties such as SSA, crystallinity, XRD size and total density of electron traps [23]. This tendency was also observed for the present samples, especially for activity for the CO_2_ system as shown in Figure 5b as a function of AR, since AR seems to be a slightly better measure for content of OAPs as shown in Figure 3d. The plots (not shown) of photocatalytic activities in both CO_2_ and H_2_ systems as a function of SSA showed trends, increase with SSA, but had poor linearity. Thus, the activity of OAP-containing samples seems to be regulated by the shape of particles. The meaning of this shape-dependent activity and another dependence of activity in the H_2_ system will be discussed later.

### 2.4. Time-Resolved Microwave Conductivity

**Figure 6:**
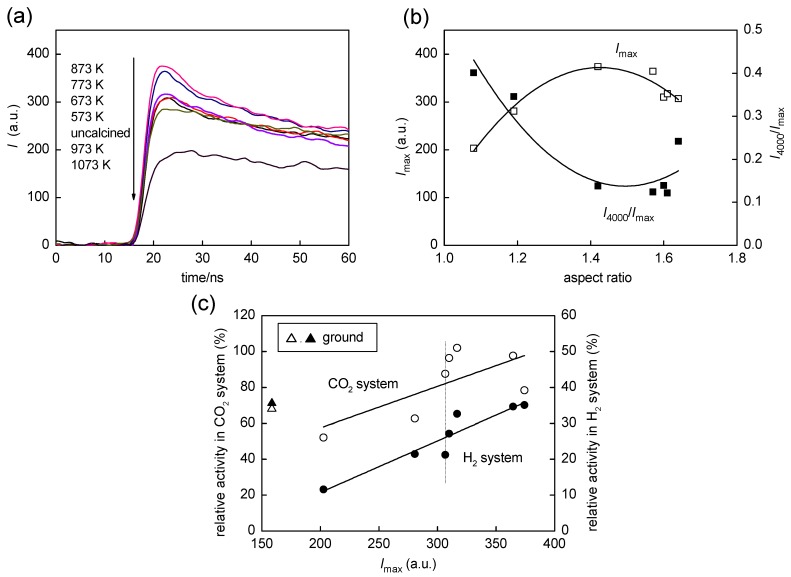
(**a**) Influence of calcination temperature on TRMC signal; (**b**) *I*_max_ and *I*_4000_/*I*_max_ as functions of AR; (**c**) Correlation between photocatalytic activities in CO_2_ and H_2_ systems and maximum intensity of the TRMC signal (*I*_max_). Data for the original samples are plotted on dotted lines. Open (CO_2_) and closed (H_2_) triangles are of the ground sample.

As one of the physical properties closely related to photocatalytic activity, time-resolved microwave conductivity (TRMC) was measured for the present samples. Such carrier dynamics may be also studied by photocurrent measurements as was reported previously [33], but due to the possible complexity in interpretation of the measurements owing to the change in sample structural properties during the electrode preparation process, we used TRMC measurement, which can be performed in the form of powder. Intensity of the TRMC signal generally shows microwave absorption by migration of charge carriers and is considered to be a product of charge-carrier density and their mobility [34]. It is also thought that positive holes, one of the charge carriers, generated in titania particles, are quickly trapped in certain sites within the time of a nanosecond laser pulse to result in negligible migration. Therefore, the TRMC signal may reflect only migration of photoexcited electrons [34]. Figure 6 shows parts of time-course curves of TRMC signals; all samples exhibited a rise of the signal within a 355-nm laser-pulse duration (*ca*. 10 ns), and decay was observed in the µs time region. First, we assume that electrons photoexcited in a conduction band (CB) are trapped in traps, the energy level of which is lower than the bottom of the CB, within a ps time scale [35], *i.e.*, faster than a process detected in TRMC measurement. Then, charge migration giving maximum signal intensity (*I*_max_) can be assigned to electron migration through the trap-detrap mechanism, *i.e.*, trapping in shallow traps and detrapping to the CB thermally, since electron hopping between deep traps is very slow [36] and thereby electrons in deep traps may have little involvement in the TRMC response. In such a circumstance, the higher the density of shallow traps is, the higher the mobility is. On the basis of these considerations, density of electrons trapped in shallow traps was slightly increased by calcination at <873 K and then decreased by calcination at >873 K. On the other hand, decay of the signal 10–20 ns after a laser pulse is attributable to trapping of electrons (once trapped in shallow traps) in deep traps, prohibiting further migration of electrons. The rate of this decay was evaluated, for convenience, by calculating the ratio of intensities at 4000 ns and the maximum (*I*_4000_/*I*_max_). The decay became slightly faster with calcination at ≤873 K, suggesting an increase in density of deep traps presumably caused by particle sintering, but, overall, the trends of *I*_max_ and *I*_4000_/*I*_max_ were of mirror images, *i.e.*, the higher *I*_max_ was, the lower was *I*_4000_/*I*_max_, indicating that the rate of trapping by deep traps depends also on the density of shallow traps.

Figure 6c shows the dependence of photocatalytic activities for CO_2_ and H_2_ systems on *I*_max_. A fairly linear relation was observed for activity in the H_2_ system. In the H_2_ system, platinum was in-situ deposited as a catalyst for hydrogen liberation by photoexcited electrons, and it has been observed that deposition of platinum reduces, in a short time region, the density of trapped electrons by capturing them [37]. Therefore, it seems reasonable that there is such a linear relation between activity in the H_2_ system and *I*_max_ with the assumption that electrons that are able to be trapped in shallow traps migrate efficiently to platinum deposits to liberate hydrogen without being trapped by deep traps. On the other hand, oxygen as a possible electron acceptor in the CO_2_ system is known to have a negligible influence on the nanosecond transient behavior of photoexcited electrons [38] and this is one of the reasons for the absence of a clear correlation with the TRMC data. Although the intrinsic reason is still ambiguous, the activity for the CO_2_ system was governed by the particle morphology as is represented by AR (Figure 5b).

### 2.5. Influence of Grinding on Structure and Photocatalytic Activity of OAP-Containing Particles

**Figure 7:**
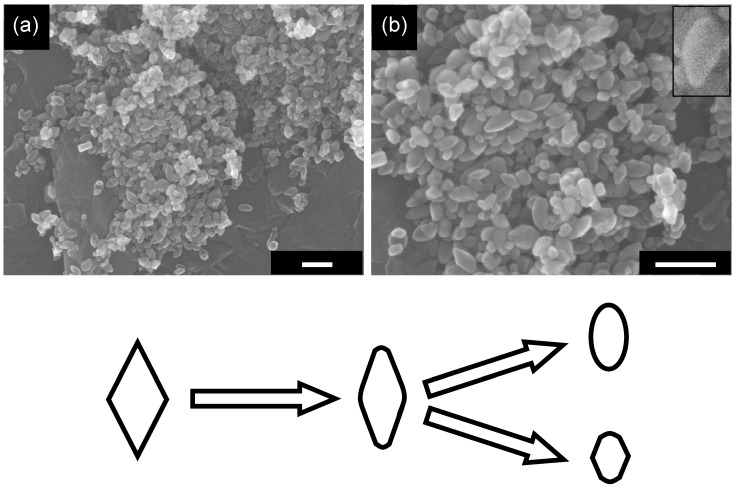
SEM images with (**a**) low magnification and (**b**) high magnification of the ground OAP-containing sample (scale bar: 100 nm) and schematic representation of change in morphology.

Grinding is one of the most commonly used post-treatment operations to make samples homogeneous by separating loosely bound agglomerates. However, grinding may also change the structure of samples and their photocatalytic activity. In this regard, the influence of grinding was studied for OAP-containing samples. After 1 h grinding in an agate mortar, the morphology of OAP-containing samples was obviously changed to round-edged as shown in Figure 7; OAPs were changed to semi-OAPs and others, as schematically shown in Figure 7.

This SEM observation is consistent with the decrease in AR (1.33) from that of the original sample (1.64) as shown in Figure 3d. (A deviation of a plot for the ground sample from a linear relation in Figure 3d might be caused by the arbitrary property of counting the numbers of semi-OAPs and others in SEM images; 30% semi-OPA content is expected from the plots.) Photocatalytic activities in CO_2_ and H_2_ systems are plotted in Figure 5b,c, respectively. Again, the activity in the CO_2_ system of the ground sample seems to be a linear relation of activity with AR (Figure 5b) but not being explained by *I*_max_ dependence (Figure 6c). On the other hand, the activity in the H_2_ system was not plotted in a linear relation with *I*_max_ (Figure 6c). It has been observed that grinding titania particles in an agate mortar for a long time (>24 h) caused the formation of deep traps, leading to negligible photocatalytic activity presumably due to enhanced recombination at the deep traps [39]. Assuming that similar deep traps were also produced in the present case with grinding for a relatively short time, decreases in *I*_max_ and *I*_4000_/*I*_max_ evaluated in TRMC measurements were accounted for by fast trapping in such deep traps in the ground sample. However, loaded platinum in the H_2_ system may capture photoexcited electrons before being trapped in the deep traps, resulting in unexpectedly higher photocatalytic activity of the ground sample in the H_2_ system. Of course, the possibility that preferable platinum loading on the ground sample led to higher photocatalytic activity in the H_2_ system cannot be excluded. Study to find the true activity-controlling structural property is now in progress.

## 3. Experimental Section

### 3.1. Preparation of OAPs Samples

Partially proton-exchanged potassium titanate nanowires (TNWs, 267 mg) were ultrasonically dispersed in Milli-Q water (40 mL) for 1 h at 298 K. The suspension was placed in a sealed Teflon bottle (100 mL), into which was poured an additional 40 mL of Milli-Q water. The bottle was heated for 24 h at 433 K in an oven without agitation. After cooling, titania as white precipitate was centrifuged, washed with RO (reverse osmosis) water, and dried under vacuum (353 K, 12 h). The thus-obtained OAP-containing samples were used as the original starting samples for further studies.

Two post-treatment operations, calcination and grinding in air, were performed. For calcination, a 1.2-g sample in an air-open ceramic crucible was placed in an oven, and the temperature was raised to a given temperature (573–1173 K) at the rate of 10 K·min^−1^, kept at that temperature for 2 h, and cooled down to ambient temperature. For grinding, a 1.2 g sample was ground in an agate mortar for 1 h in air.

### 3.2. Characterization

Specific surface area of samples was evaluated by nitrogen adsorption at 77 K using the Brunauer–Emmett–Teller (BET) equation. The morphology was studied by scanning electron microscopy (SEM, JEOL JSM-7400F, Akishima, Japan), scanning transmission electron microscopy (STEM, HITACHI HD-2000, Tokyo, Japan) and transmission electron microscopy (TEM, JEOL JEM-2100F). Particles in a sample were classified into three groups based on the results of SEM analysis: (a) OAP, an octahedral particle without observable defects; (b) semi-OAP, an octahedral particle with a defect (defects) and (c) others, an irregular shaped non-octahedral particle. Composition of these particles in each sample was measured by counting at least 200 particles in several SEM images of a sample [23].

XRD analysis was performed using the SmartLab intelligent X-ray diffraction system (Rigaku, Akishima, Japan) equipped with a sealed tube X-ray generator (a copper target; operated at 40 kV and 30 mA), a D/teX high-speed position-sensitive detector system and an ASC-10 automatic sample changer. Data acquisition conditions were as follows: 2θ range, 10–90°; scan speed, 1.00°·min^−1^; and scan step, 0.008°. The obtained XRD patterns were analyzed by Rigaku PDXL, a crystal structure analysis package including Rietveld analysis [40], installed in a computer controlling the diffractometer. Crystallite size (XRD size) was evaluated from corrected width of an anatase 101 diffraction peak using the Scherrer equation. Crystallinity of a sample was evaluated using an internal standard, highly crystalline nickel oxide (NiO). The standard (20.0 wt %) was mixed thoroughly with a sample (80.0 wt %) by braying in an agate mortar. Since Rietveld analyses give composition of each crystal among total crystal content, the composition of the standard (formally 20.0 wt %) is measured to be larger if the sample contains a non-crystalline component. Therefore, crystalline and non-crystalline compositions are estimated by re-calculation to make the standard composition to be 20.0 wt %. At present, the authors regard the non-crystalline part to be water, which can be estimated by thermogravimetry, and amorphous titania and/or titanates.

### 3.3. Photocatalytic Activity Test

Photocatalytic activities of samples were examined by measuring the amount of evolved carbon dioxide (CO_2_) and hydrogen (H_2_) from continuously stirred (1000 rpm) suspensions of a sample (50 mg) in an aerated aqueous acetic acid solution (5.0 mL, 5.0 vol %) (CO_2_ system) and a deaerated aqueous methanol solution (5.0 mL, 50 vol %) containing chloroplatinic acid (corresponding to 2.0 wt % (as platinum) of a photocatalyst) for *in-situ* platinum photodeposition (H_2_ system), respectively. Photoirradiation (>290 nm) was performed with a 400-W high-pressure mercury lamp (Eiko-sha, Osaka, Japan) at 298 K. Amounts of liberated CO_2_ and H_2_ in gas phase were measured by gas chromatography (TCD-GC). The photocatalytic activities are presented with reference to those of a commercial titania photocatalyst, Showa Denko Ceramics FP-6 (anatase, SSA: *ca*. 100 m^2^·g^−1^, XRD size: 15 nm). FP-6 is known to exhibit a high level of photocatalytic activity among commercial titania powders, similar to well-known Evonik (Degussa, Essen, Germany) P25 [41,42]. The average rates of FP-6 and P25 were, respectively, *ca*. 0.54 and 0.91 µmol·h^−1^ in the H_2_ system and 0.039 and 0.046 µmol·h^−1^ in the CO_2_ system [23]. Due to negligible solubility of H_2_ in water and CO_2_ in an aqueous solution of acetic acid, no correction was made for gas dissolution in reaction suspensions.

### 3.4. Time-Resolved Microwave Conductivity Measurements

Charge-carrier dynamics was studied by measuring time-resolved microwave conductivity (TRMC) induced by a ns time-scale laser pulse in Laboratory of Physical Chemistry, University of Paris-Sud. Incident 36.8-GHz microwaves and UV laser pulses were generated by a Gunn diode of the K_a_ band and third harmonic of a 1064-nm Nd:YAG laser (10 Hz) with full width at half maximum of *ca.* 10 ns, respectively [43].

## 4. Conclusions

Post-treatments, calcination and grinding, of OAP-containing particles changed structural properties, including OAP/semi-OAP content, aspect ratio (AR) and particle size, and TRMC responses, e.g., *I*_max_ and *I*_4000_/*I*_max_ reflecting change in charge-carrier dynamics. The changes caused by calcination were interpreted by annealing of single-crystal OAPs and sintering to be aggregates of single crystals of OAPs, leading to, respectively, higher crystallinity/higher density of shallow traps and slightly lower crystallinity/lower aspect ratio, *i.e.*, lower OAP content/higher density of deep traps due to grain boundaries, while the changes caused by grinding were interpreted by round-edging,*i.e.*, decrease in OAP (and semi-OAP) content/higher density of deep traps. As a result, photocatalytic activities in CO_2_ and H_2_ systems were linearly increased or decreased depending on AR reflecting OAP content and maximum TRMC signal intensity (*I*_max_), respectively. Although few supporting experimental results have been obtained, a working hypothesis is that photocatalytic activity is governed by electron traps, the density of which is influenced by the method of preparation and post-treatment [44], and that most electron traps are located on the surface of particles and their energy depends on the structure of exposed surfaces such as {101} facets on OAPs. Analyses of energy-resolved density of electron traps [45] in the present OAP-containing particles are now in progress and results will be published in the near future.
